# Predicting Response to Immune Checkpoint Inhibitors in Melanoma: Emerging Approaches in Digital Pathology, Spatial Profiling and Machine Learning

**DOI:** 10.3390/ijms27125244

**Published:** 2026-06-10

**Authors:** Jakub Banaszek, Dawid Bąk, Kinga Barańska, Alicja Czajka, Dominika Ciesielska, Jakub Kleinrok, Weronika Pająk, Agnieszka Korolczuk, Maciej Mazur, Kamil Rusztyn

**Affiliations:** 1 Chair and Department of Clinical Pathomorphology, Medical University of Lublin, Jaczewskiego 8b, 20-090 Lublin, Poland; kubabanaszek40@gmail.com (J.B.); dawidbak123321@gmail.com (D.B.); kinga14b@gmail.com (K.B.); alaczajka2003@gmail.com (A.C.); dciesielska600@gmail.com (D.C.); klejs.90@gmail.com (J.K.); wapajak@gmail.com (W.P.);; 2 Faculty of Chemistry, University of Warsaw, Ludwika Pasteura 1, 02-093 Warsaw, Poland; 3 Faculty of Medicine, Medical University of Warsaw, Ul. Zwirki i Wigury 61, 02-091 Warsaw, Poland

**Keywords:** melanoma, digital pathology, machine learning, multiplex methods, immune checkpoint inhibitors (ICIs), response predictors

## Abstract

The introduction of immune checkpoint inhibitors (ICIs) into the treatment of melanoma has significantly reduced mortality over the past decade. However, therapeutic benefit is not observed in all patients, and treatment may be associated with severe adverse events. Therefore, identifying patients who are most likely to benefit from immunotherapy remains of critical importance. Currently used biomarkers, such as programmed death-ligand 1 (PD-L1) expression and manual assessment of tumour-infiltrating lymphocytes (TILs), have limited predictive value. This narrative review provides a critical appraisal of studies employing digital pathology tools, multiplex and spatial techniques (including multiplex immunofluorescence, imaging mass cytometry, and digital spatial profiling), as well as machine learning algorithms for predicting response to ICIs in patients with melanoma. Available evidence suggests that the highest predictive value may be achieved by approaches integrating quantitative assessment of immune infiltration with information on its spatial distribution, functional state, and interactions within the tumour microenvironment. Particular relevance may be attributed to features associated with the “immune-inflamed”, “immune-excluded”, and “immune-desert” phenotypes, the presence of tertiary lymphoid structures, and the organisation of local immune niches. In addition, this review highlights key limitations in the interpretation of current data, including lack of methodological standardisation, data heterogeneity, and insufficient validation. Directions for future research necessary for the implementation of these approaches into routine clinical practice are also discussed.

## 1. Introduction

### 1.1. Melanoma as a Global Health Problem

Melanoma is a malignant neoplasm arising from melanocytes located in the basal layer of the epidermis [1]. Although it accounts for approximately 1% of all skin cancers, it is responsible for nearly 80% of skin cancer-related deaths, primarily due to its high metastatic potential and resistance to conventional treatment modalities [1,2].

The incidence of melanoma continues to rise; projections indicate that by 2040, global incidence will increase by approximately 50% compared with 2020, reaching nearly half a million new cases annually [3]. This trend is associated with population ageing, increased exposure to ultraviolet radiation, and intensified diagnostic activity, including the phenomenon of overdiagnosis [3,4]. Despite these factors, the global health burden of melanoma remains substantial [3].

### 1.2. Immunological Determinants of Response to ICIs

Advances in immuno-oncology have substantially improved outcomes in patients with melanoma. The introduction of immune checkpoint inhibitors (ICIs), including anti-programmed cell death protein 1 (anti-PD-1), anti-cytotoxic T-lymphocyte-associated protein 4 (anti-CTLA-4), and anti-lymphocyte-activation gene 3 (anti-LAG-3) antibodies, as well as targeted therapies, has been associated with a decline in melanoma mortality in the United States by nearly 6% per year during 2013–2017, followed by a further decrease of 1.2% per year during 2017–2023 [5]. However, treatment efficacy depends on the tumour immune phenotype, and appropriate patient selection is critical given the risk of severe adverse events [6,7,8,9].

The immune phenotype reflects the state of the tumour microenvironment (TME), which comprises tumour, immune, and stromal cells. Their interactions determine the response to ICIs. T-lymphocytes, particularly cytotoxic CD8^+^ T-cells, play a central role, and high levels of infiltration are generally associated with improved prognosis [10]. In clinical practice, not only the extent of infiltration but also the functional state of these cells (activation versus exhaustion) and the expression of inhibitory receptors are crucial, as they may determine sensitivity or resistance to ICIs [11].

Based on the distribution and activity of immune cells, three major phenotypes are distinguished: immune-inflamed, immune-excluded, and immune-desert [8]. Immune-inflamed (“hot”) tumours are characterised by abundant T-cell infiltration within the tumour parenchyma, activation of the interferon-γ (IFN-γ) pathway, increased programmed death-ligand 1 (PD-L1) expression, and often elevated tumour mutational burden (TMB). These tumours typically show better responses to ICIs and are associated with improved overall survival [12]. Increasing attention is also being paid to the role of B-cells and tertiary lymphoid structures (TLSs), which may contribute to the formation of “hot” immune niches and correlate with improved therapeutic response [13,14].

The immune-excluded phenotype is characterised by the presence of immune cells in the peritumoural stroma without effective infiltration into the tumour core. This condition is associated with disrupted chemokine signalling, activation of oncogenic pathways, hypoxia, abnormal angiogenesis and stromal barriers within the TME [12]. One of the mechanisms leading to the ‘immune-excluded’ phenotype is increased tumour stromal activity dependent on transforming growth factor beta (TGF-β) signalling. In particular, TGF-β acts on cancer-associated fibroblasts (CAFs), promoting fibrosis and extracellular matrix remodelling, including collagen deposition and organisation. This stromal architecture favours the retention of lymphocytes in the peritumoural stroma rather than their penetration into the tumour parenchyma. Consequently, response to ICI monotherapy in this group of patients is limited [15,16].

**Figure 1:**
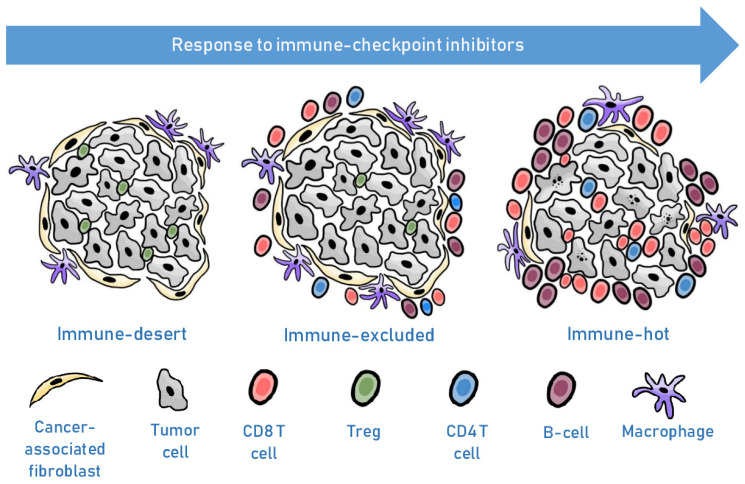
Three tumour immune phenotypes can be distinguished based on the distribution and activity of immune cells: immune-desert, immune-excluded and immune-inflamed. Their response to immune checkpoint inhibitors (ICIs) generally increases in this order, with immune-desert tumours showing the weakest and immune-inflamed tumours the strongest response.

Immune-desert (“cold”) tumours are characterised by a lack of effective priming and minimal T-cell infiltration. They typically exhibit low TMB, reduced expression of major histocompatibility complex class I (MHC I) molecules and PD-L1, impaired antigen presentation, and a predominance of immunosuppressive mechanisms, including regulatory T-cells (Tregs), myeloid-derived suppressor cells (MDSCs), and tumour-associated macrophages [10,12]. Immune-desert tumours generally show poor response to ICIs [12]. A schematic representation of the tumour immune phenotypes is shown in Figure 1.

In summary, melanoma resistance to ICIs is a multifaceted process in which effective antitumour immunity may be impaired at multiple levels. Mechanisms of resistance may arise from tumour cell-intrinsic alterations, defects in antigen presentation and recognition, impaired recruitment or trafficking of immune cells, their spatial organisation within the tumour, the immunosuppressive properties of the TME, and functional dysfunction or exhaustion of effector lymphocytes [10,11,12,13,14,15,16].

### 1.3. Why Are Standard Histopathological Techniques Insufficient for Predicting Response to ICIs?

Routine histopathological assessment of melanoma provides important information regarding disease stage and prognosis; however, its usefulness in predicting response to ICI treatment remains limited [17,18]. Standard parameters, such as characteristics of the primary tumour, regional lymph node involvement or the presence of distant metastases, are primarily of prognostic significance and do not fully reflect the complexity of the TME, which significantly determines the efficacy of immunotherapy [12,17,18].

In histopathological practice, one of the most frequently analysed indicators of immune response is tumour-infiltrating lymphocytes (TILs), assessed mainly in haematoxylin and eosin (H&E)-stained sections, as well as using immunohistochemistry (IHC) [19]. Manual quantitative assessment of stromal lymphocytes as a percentage in the invasive part of the tumour has also been proposed as a way to standardise TIL evaluation [20]. Immunohistochemistry additionally allows for the identification of selected subpopulations of immune cells, such as CD3+, CD4+, CD8+ or FOXP3+ lymphocytes, enabling a more detailed characterisation of the TME [17,19].

However, these approaches have significant limitations. Manual assessment of TILs remains partly subjective and is prone to inter-observer variability [21]. Furthermore, it does not fully capture the spatial relationships between tumour cells, immune cells and the stroma. Similarly, the measurement of individual biomarkers, such as PD-L1, does not provide sufficiently precise patient selection for ICI therapy, owing to the limited predictive value of this marker and its dynamic expression [22].

For this reason, tools of digital and spatial pathology are gaining increasing importance, as they enable a more objective, quantitative and multidimensional characterisation of the tumour microenvironment [14,23,24,25,26,27,28,29,30,31,32]. The aim of this study is to assess their utility in predicting response to ICIs in melanoma patients and to discuss the most significant methodological limitations of the available studies.

## 2. Literature Review

This paper is a narrative review that provides a concise overview of the most important and representative studies on predicting response to immune checkpoint inhibitors in melanoma patients using digital pathology, spatial methods and machine learning. The literature review was conducted using the PubMed and Google Scholar databases, focusing primarily on peer-reviewed English-language publications published between 1 January 2019 and 1 April 2026. Particular attention was paid to original studies concerning new predictive biomarkers, methods integrating imaging and spatial data, and methodological limitations affecting the clinical implementation of these approaches.

The database search used combinations of the following keywords: “melanoma”, “tumour microenvironment”, “tumour-infiltrating lymphocytes”, “digital pathology”, “machine learning”, “multiplex methods”, “Digital Spatial Profiling”, “immunohistochemistry”, “immune checkpoint inhibitors”, and “response predictors”. In addition, the reference lists of selected publications were manually screened to identify relevant studies that may not have been captured during the initial search.

The review primarily included original studies investigating predictors of response to ICIs in melanoma, with particular emphasis on tissue-based biomarkers, H&E image analysis, immunohistochemistry, multiplex techniques, spatial profiling, and machine learning models. Selected review articles were also included when they provided relevant background on the biological basis of ICI response, interpretation of the tumour microenvironment, or translational challenges. Publications focused exclusively on other cancer types were considered only when they provided information relevant to broader methodological or technological issues.

Non-peer-reviewed publications, conference abstracts without a full-text article, non-English publications, studies unrelated to melanoma, and articles lacking data relevant to ICI response prediction or the interpretation of digital and spatial pathology methods were excluded. Given the narrative nature of this review, a formal PRISMA-based study selection process was not performed, and no flow diagram was generated. Consequently, the total number of identified and screened records was not reported, which should be considered a limitation of this review.

Publication selection was performed by the authors based on thematic relevance, methodological quality, and significance to the topics discussed. In cases of uncertainty, inclusion decisions were made following discussion and consensus among the authors.

## 3. Digital Pathology and Spatial Profiling of TME

### 3.1. Quantitative Assessment of TILs and Other Immune Infiltrates in Digital Images

Previous studies suggest that quantitative assessment of immune infiltration in digital images may be one of the most promising and also practical tools for predicting response to ICIs in melanoma patients. These approaches are most often based on the analysis of whole-slide images (WSIs) of H&E or IHC-stained slides, using machine learning algorithms to automatically quantify TILs [23,24,25].

A similar conclusion emerges from the studies by Chatziioannou et al. (2023) [24], Schuiveling et al. (2025) [25] and Fortman et al. (2024) [23]. Despite methodological differences, all three studies indicate that a higher level of lymphocytic infiltration assessed digitally is associated with a better response to anti-PD-1 treatment [23,24,25]. Chatziioannou et al. showed that low electronic tumour-infiltrating lymphocyte score (eTILs), reflecting the proportion of lymphocytes relative to tumour cells in H&E image analysis, may be associated with poorer treatment efficacy in patients with metastatic melanoma who had not received prior systemic treatment [24]. Similarly, Schuiveling et al. showed that a higher percentage of TILs assessed by an artificial intelligence (AI) algorithm correlates with a better treatment response, and that this relationship was more pronounced than with manual assessment [25]. Fortman et al., in turn, noted that absolute T-cell density measured on whole-slide images using CD8 staining may also have predictive significance [23]. Taken together, the results of this work suggest that digital TIL assessment may be superior to manual assessment in terms of reproducibility and standardisation potential, although different definitions of TILs and the need for further validation of proposed thresholds and indices remain important limitations [23,24,25]. The main results and methodological differences between studies are presented in Table 1.

In addition, Vaňková et al. (2026) proposed a broader approach, analysing tumour-infiltrating immune cells (TIICs), including T-lymphocytes (CD3), cytotoxic lymphocytes (CD8), dendritic cells (CD1a) and expression of LAG3, PD1 and PD-L1 markers in patients with metastatic melanoma [33]. Despite methodological differences, the results were partly consistent with previous observations, as a higher infiltration of CD8+ cells was also associated with a significantly better response to ICIs [23,24,25,33]. Similar significant relationships were shown for other parameters of the tumour microenvironment, including greater infiltration of PD-L1-positive cells and lower infiltration of CD1a-positive cells. This suggests that TIICs may provide additional predictive value compared to TILs [33].

Together, these results indicate that the predictive value of digital slide analysis is not solely dependent on the number of immune cells, but probably also on their type and organisation within the TME, justifying the growing interest in methods that take into account spatial relationships within the tumour [23,24,25,33].

### 3.2. Spatial Profiling of the Tumour Microenvironment

#### 3.2.1. Spatial Proteomics

Currently, an increasing number of studies on predicting ICI response in melanoma patients focus on integrated TME analysis rather than single-marker assessments. Of particular relevance are methods based on immunohistochemistry and fluorescence, especially quantitative immunofluorescence (QIF) and multiplex immunofluorescence (mIF), which, together with machine learning, enable simultaneous assessment of the cellular composition of the immune infiltrate, its localisation and spatial relationships between cells [14,26,27].

One early study using these methods suggested that quantitative assessment of the infiltrate alone may have predictive value. Wong et al. (2019) showed that machine learning-determined TILs and QIF-determined CD4, CD8 and CD20 protein counts correlated positively with response to anti-PD-1 therapy, particularly for CD8 lymphocytes [26]. Similarly, Gide et al. (2019), using mIF, showed that response to anti-PD-1 treatment was associated not only with the presence of immune cells but also with their close localisation to tumour cells, further highlighting the importance of the spatial architecture of the TME [34]. In addition, subsequent studies suggest that the integration of these parameters with spatial information may have greater predictive value than single molecular features [14,27,35].

Pybus et al. (2026) [27], using mIF and machine learning, showed that neither TILs, TME cellular composition nor nitric oxide synthase levels alone could predict response to anti-PD-1 therapy. The best results were obtained when all parameters were combined, especially in areas rich in immune cells. In responding patients, TIL niches were enriched in CD8, LAG-3, PD-L1 and SOX10 [27]. PD-1 and LAG-3 are jointly involved in T-cell depletion, and the clinical relevance of their simultaneous blockade has been confirmed in studies of relatlimab and nivolumab [36,37]. The authors also demonstrated an association between higher inducible nitric oxide synthase (iNOS) expression and better response to ICIs, although iNOS was previously considered a prognostically unfavourable factor, which may be due to the concentration-dependent activity of this enzyme. An important factor limiting the translation of these findings into clinical practice is the small sample size of the study (n = 12) [27].

The importance of organised local immune niches has also been suggested by studies involving B-cells and TLS. Smithy et al. (2025), using mIF and a spatial clustering algorithm, showed that a higher percentage of B-cells in the tumour stroma and the presence of quantitatively defined B-cell aggregates were associated with response to ICIs in melanoma patients [14]. These findings are consistent with previous observations by Griss et al. (2019) and Helmink et al. (2020), which indicated the beneficial role of B-cells and TLS in response to ICIs in melanoma [38,39]. This is likely due to their role in local priming and the maintenance of T-cell responses in the TME. At the same time, the lack of standardised criteria for assessing TLS remains an important translational limitation [14,40]. Importantly, in the study by Smithy et al. (2025) [14], the presence of lymphoid aggregates assessed by the pathologist was not associated with response to ICIs, in contrast to computational analysis. This result suggests that not only the presence of lymphoid aggregates, but also the way in which they are quantified, may influence the value of TLS as a biomarker (Table 1) [14].

The relationship between T-lymphocytes and dendritic cells may also be important in predicting response to ICIs treatment. Gobbini et al. (2025) showed that closer proximity and more frequent interactions between CD8+ T-cells and conventional type 1 dendritic cells (cDC1s) were associated with better treatment outcomes, whereas no such relationship was observed for CD8+ T-cell numbers alone [35]. Similar findings were reported by Pybus et al. (2026), indicating that treatment response depends not only on the abundance of effector cells, but also on their spatial organisation and interactions within the TME [27,35]. Additional studies suggest that specific dendritic cell subtypes, particularly mature dendritic cells with an immunoregulatory phenotype (mregDCs), may also be associated with response to ICIs. This relationship has been observed even in patients treated with combined anti-CTLA-4 and anti-PD-1 therapy [41].

Another approach used in spatial proteomics is imaging mass cytometry (IMC), which allows for the simultaneous assessment of multiple markers together with their spatial distribution in the tumour microenvironment [42,43,44]. Using this technique, Xiao et al. (2022) [42] identified six distinct TME archetypes and showed that they differed in their response to anti-PD-1 treatment. Immune-hot archetypes were associated with the highest response rates, but responders were not a biologically homogeneous group and only some tumours showed high CD8+ infiltration before treatment. In contrast, immune-cold archetypes, including variants with tumour cell predominance, hypoxia or suppressive myeloid cells, were associated with low response rates [42]. It should be noted, however, that the external validation primarily concerned the 24-gene transcriptomic score derived from differential gene expression between immune-hot and immune-cold tumours. Therefore, the predictive value of the IMC-defined archetypes themselves requires further validation [42].

Similarly, Van Dam et al. (2025), using IMC, reported that tumours from responding patients contained more monocyte-derived macrophages (MDMs) and cytotoxic T-cell subsets, whereas tumours from non-responders more often showed co-localisation of suppressive M2-type macrophages with T-lymphocytes [43]. These observations are consistent with other studies indicating the importance of the CXCR3 axis and increased macrophage secretion of the chemokines CXCL9 and CXCL10, which promote CD8+ T-cell influx into the tumour and improve the efficacy of ICI therapy; indeed, Van Dam et al. also reported significantly elevated messenger RNA (mRNA) levels of these chemokines [43,45]. In addition, more recent data suggest that inflammatory monocytes may also have a similar function, directly promoting CD8+ T-cell stimulation within the tumour through the expression of CXCL9, CXCL10 and IL-15 and the presentation of class I peptide–MHC complexes. This suggests that the efficacy of immunotherapy depends not only on the presence of myeloid cells, but also on their activation state and ability to sustain a local anti-tumour response [46]. However, it should be emphasised that the study by Van Dam et al. was based on a small cohort of only 14 patients. Consequently, the observed association between MDMs and response to anti-PD-1 therapy should be interpreted with caution and requires validation in larger, independent cohorts [43]. Consistent results were reported by Giuliani et al. (2024), who combined publicly available IMC data with a spatial mechanistic model to assess which features of the TME might influence response to immunotherapy [47]. They showed that the density of cell populations alone poorly discriminated between responders and non-responders, whereas the spatial organisation of the TME, particularly the relationship between activated CD8+ lymphocytes and macrophages, was more important. The model further indicated the importance of CD8+ T-cell exhaustion and the “fencing” phenomenon, involving the accumulation of exhausted lymphocytes at the tumour boundary, which may restrict access of activated effector cells to tumour cells and promote progression. These results support the conclusion that response to ICIs depends not only on the cellular composition of the TME, but also on its spatial architecture and the dynamics of cellular interactions [47].

Another method categorised as spatial proteomics is Digital Spatial Profiling (DSP), performed using the GeoMx platform. This method involves selecting regions of interest (ROIs) in a tissue section and performing a high-plex readout of proteins or RNAs from these regions to analyse signals in specific tumour compartments [28,29]. Using this method, Toki et al. (2019) [28] showed that the strongest predictive marker of ICI benefit was PD-L1 expression in CD68+ cells, but not in tumour cells. Moreover, the CD8 signal assigned by the algorithm to the CD68+ compartment also had predictive value, likely reflecting spatial proximity between CD8+ lymphocytes and macrophages [28]. A similar conclusion emerged from the study by Martinez-Morilla et al. (2023) [29], in which high PD-L1 expression in the stroma was associated with better response to ICIs, whereas CD95 expression in specific tumour compartments and CD68 expression were associated with treatment resistance. This finding suggests that CD95 may represent a negative predictive marker, although its biological role remains complex [29].

In conclusion, the results of the cited studies using QIF, mIF, IMC and DSP methods suggest that prediction of response to ICIs in melanoma should not rely solely on the assessment of single markers or the extent of lymphocytic infiltration, but rather on a more comprehensive analysis of the TME, taking into account specific cellular niches, TME archetypes and spatial relationships between immune and tumour cells [14,26,27,28,29,35,43].

**Table 1:** Selected studies on digital and spatial pathology for predicting response to immune checkpoint inhibitor therapy in patients with melanoma.

Reference	Methods	Population/ICI	Main Outcomes	Cohort/Validation
A. Digital pathology and machine learning/AI image analysis				
Wong et al. (2019) [26]	mIF + QIF, quantitative TIL phenotyping	Metastatic melanoma, anti-PD-1, n = 94	Quantitative TIL metrics were positively associated with ICI response CD8 predictive performance: AUC > 0.75 for ORR/DCR; up to 0.83 in ipilimumab + nivolumab	Single cohort; no external validation
Hu et al. (2021) [30]	DL on H&E WSI	Advanced melanoma, anti-PD-1 monotherapy, test cohort n = 54	H&E-based deep learning predicted anti-PD-1 RECIST 1.1-defined response with an AUC of 0.778 Predicted responders had longer PFS The model outperformed TIL-based prediction of anti-PD-1 response (AUC 0.58)	Single cohort; independent melanoma test set; cross-cancer test in NSCLC
Johannet et al. (2021) [32]	H&E whole-slide imaging + DCNN, integrated with clinical variables in a multivariable logistic model	Advanced melanoma, first-line ICI (anti-CTLA-4, anti-PD-1, or combination), training n = 121, validation n = 30	The integrated classifier predicted ICI response vs. progression by RECIST 1.1 with AUCs of 0.800 (Aperio AT2) and 0.805 (Leica SCN400) in the validation cohort DCNN-only performance was lower (AUCs of 0.707 and 0.667), and prediction was stronger in lymph node than in soft-tissue metastases	Single training cohort; independent external validation cohort
Fortman et al. (2024) [23]	Automated IHC CD8 quantification (WSI)	Advanced melanoma, anti-PD-1, n = 78	Higher CD8+ TIL density was associated with better response A cut-off ≥ 222.9 cells/mm^2^ discriminated responders from non-responders and was associated with improved PFS, with a trend towards improved OS	Single cohort; no external validation
Fa’ak et al. (2025) [31]	H&E whole-slide imaging + supervised and self-supervised AI	Stage III/IV melanoma on ICI [anti-CTLA-4 (n = 212), anti-PD-1 (n = 271), combination (n = 156)], total n = 639; external CheckMate-067 n = 196	The supervised model predicted RECIST-defined ICI response in metastatic melanoma with an AUC of 0.72 The DCNN stratified patients into high- vs. low-risk groups with significantly different PFS Self-supervised analysis identified epithelioid histology and low tumour–stroma ratio as features associated with better survival after ICI	Multicentre/international cohorts including trials; external validation in CheckMate-067
Schuiveling et al. (2025) [25]	AI-based TIL% quantification	Advanced melanoma, anti-PD-1 ± anti-CTLA-4, n = 1202	Higher AI-detected TILs were associated with better ICI response and longer PFS/OS Each 10% increase in TILs was associated with higher ORR AI-detected TILs showed stronger predictive value than manual TIL scoring	Large multicentre cohort; no external validation
Vaňková et al. (2026) [33]	Stereology + IHC	Metastatic melanoma on ICI, n = 28 (response analysis in n = 20)	Higher PD-L1 infiltration predicted better iRECIST-defined ICI response in all metastases (AUC 0.66) In lymph node metastases, higher PD-L1 (AUC 0.7704) and CD8+ (AUC 0.75), and lower CD1a+ (AUC 0.7535), predicted better iRECIST-defined ICI response Lower PD-L1 expression and lower CD3 expression were associated with irAEs	Single cohort; no external validation
B. Spatial/multiplex/multiomics approaches				
Toki et al. (2019) [28]	DSP (GeoMx)	Melanoma, ICI, n = 60	PD-L1 in CD68+ macrophages, not tumour cells, predicted better immunotherapy response, PFS and OS High CD8 in the CD68+ compartment was associated with better response and longer PFS/OS	Single cohort; DSP validated against QIF and by inter-core reproducibility
Cabrita et al. (2020) [13]	TLS-associated transcriptomic signature derived from IHC/IF, with GeoMx support	Melanoma, anti-CTLA-4 (n = 37 and n = 40), anti-PD-1 ± anti-CTLA-4 (n = 69), anti-PD-1 (n = 41)	A higher TLS gene signature predicted better ICI outcomes in melanoma Predictive value was shown across anti-CTLA-4 and anti-PD-1 cohorts The TLS signature was independent of TMB	Single discovery cohort for tissue analysis; multiple independent external validation cohorts for the TLS gene signature
Xiao et al. (2022) [42]	IMC + RNA-seq-derived TME response score	Advanced melanoma, anti-PD-1 monotherapy, n = 26	Six IMC-defined TME archetypes were associated with differential RECIST 1.1-defined anti-PD-1 response: immune-hot H1 CD4+ T/B-cell-rich, H2 HLA-DR + CD11c+ myeloid-rich, H3 CD8+ T-cell-rich, and immune-cold C1 CAIX+ tumour-cell-rich, C2 HLA-DR−CAIX+ myeloid-rich, and C3 with no dominant enrichment A 24-gene TME response score predicted RECIST 1.1-defined anti-PD-1 response across cohorts (AUC 0.83, 0.75, 0.74, 0.65)	Single-centre discovery cohort; external transcriptomic validation in three independent anti-PD-1 cohorts; no external IMC validation
Martinez-Morilla et al. (2023) [29]	DSP (GeoMx)	Metastatic melanoma, anti-PD-1 and/or anti-CTLA-4, n = 53	CD95 (FAS) expression in immune cells was associated with resistance to ICIs	Discovery cohort; independent validation cohort; technical validation by QIF/mIF
Quek et al. (2024) [48]	Single-cell spatial multiomics (CITE-seq + PhenoCycler/CODEX) with multimodal integration	Metastatic melanoma, anti-PD-1 + anti-CTLA-4, n = 5	A lymphoid-aggregate-associated B-cell signature (*TNFRSF13C*, *BLK*, *CD79A*) was enriched in responders and distinguished response from resistance to ICIs A high B-cell signature was associated with longer PFS and OS in an external anti-PD-1 cohort Lymphoid aggregates located intra-tumourally were associated with response, whereas stromal/peritumoural aggregates characterised innate resistance Loss of memory-state T-cells was linked to acquired resistance	Small longitudinal discovery cohort; external transcriptomic validation of the B-cell signature in independent anti-PD-1 and anti-PD-1 + anti-CTLA-4 cohorts
Aung et al. (2024) [49]	DSP-WTA	Advanced melanoma, anti-PD-1, discovery n = 55, validation n = 45	Spatial compartment signatures predicted RECIST 1.1-defined OR better than pseudo-bulk analysis (AUC: S100B 0.86, CD68 0.94, CD45 0.98 vs. pseudo-bulk 0.70) The 8-gene S100B tumour signature showed the best external validation for RECIST 1.1-defined OR prediction (AUC 0.79) and outperformed bulk RNA signatures In the S100B signature, *PSMB8*, *TAX1BP3*, *NOTCH3*, *LCP2*, and *NQO1* predicted response, whereas *KMT2C*, *OVCA2*, and MGRN1 predicted resistance	Single cohort; independent validation; additional external validation in two published bulk RNA-seq cohorts
Pybus et al. (2026) [27]	mIF + spatial ML models	Stage IV melanoma, pre-anti-PD-1, n = 12	ML models predicted durable RECIST-defined anti-PD-1 response vs. early PD in 11/12 patients Best AUC (0.76) was achieved with combined compositional/spatial features in immune-rich regions Key features included immune composition, spatial interactions, NOS-related markers, and a TIL-like neighbourhood No single biomarker predicted response	Single cohort; no external validation
Smithy et al. (2025) [14]	mIF + quantitative spatial features	Unresectable melanoma, anti-PD-(L)1 monotherapy or anti-PD-1 + anti-CTLA-4, n = 50	Higher stromal CD20+ B-cell percentage predicted RECIST and irRC-assessed ICI response Stromal B-cell percentage predictive performance: AUC = 0.825 Predictive value remained after excluding lymph node samples (AUC = 0.772)	Single cohort; external validation in an independent anti-PD-1 RNA-seq cohort
Van Dam et al. (2025) [43]	IMC + RNA-seq validation	Stage IV metastatic non-uveal melanoma, first-line anti-PD-1 monotherapy, n = 14	Higher baseline infiltration of monocyte-derived macrophages (MDMs) and cytotoxic T-cell subsets was associated with RECIST 1.1-defined anti-PD-1 response Non-responders showed spatial co-localisation of suppressive M2 macrophages and T-cells MDM infiltration was associated with RECIST 1.1-defined response and improved PFS/OS	Single-centre cohort; external transcriptomic support for the MDM/macrophage-associated response signal

Abbreviations: AI, artificial intelligence; AUC, area under the curve; CAIX, carbonic anhydrase IX; CD1a, cluster of differentiation 1a; CD3, cluster of differentiation 3; CD4, cluster of differentiation 4; CD8, cluster of differentiation 8; CD11c, cluster of differentiation 11c; CD20, cluster of differentiation 20; CD45, cluster of differentiation 45; CD68, cluster of differentiation 68; CD79A, cluster of differentiation 79A; CD95 (FAS), Fas cell surface death receptor; CITE-seq, Cellular Indexing of Transcriptomes and Epitopes by Sequencing; CTLA-4, cytotoxic T-lymphocyte-associated protein 4; DCNN, deep convolutional neural network; DCR, disease control rate; DL, deep learning; DSP, digital spatial profiling; DSP-WTA, Digital Spatial Profiling Whole Transcriptome Atlas; H&E, haematoxylin and eosin; HLA-DR, human leukocyte antigen DR isotype; ICIs, immune checkpoint inhibitors; IHC, immunohistochemistry; IF, immunofluorescence; IMC, imaging mass cytometry; irAEs, immune-related adverse events; irRC, immune-related Response Criteria; mIF, multiplex immunofluorescence; ML, machine learning; MDMs, monocyte-derived macrophages; NOS, nitric oxide synthase; NSCLC, non-small-cell lung cancer; ORR, objective response rate; OS, overall survival; PD, progressive disease; PD-1, programmed cell death protein 1; PD-(L)1, programmed cell death protein 1/programmed death-ligand 1 axis; PD-L1, programmed death-ligand 1; PFS, progression-free survival; QIF, quantitative immunofluorescence; iRECIST, immune Response Evaluation Criteria in Solid Tumours; RECIST, Response Evaluation Criteria in Solid Tumours; RNA-seq, RNA sequencing; TILs, tumour-infiltrating lymphocytes; TMB, tumour mutational burden; TME, tumour microenvironment; TLS, tertiary lymphoid structures; WSI, whole-slide imaging.

#### 3.2.2. Spatial Transcriptomics and Multimodal Profiling

Spatial transcriptomics-based approaches complement spatial proteomics by enabling the assessment of gene expression while preserving spatial context within the TME. One example is the study by Aung et al. (2024), in which the Digital Spatial Profiling Whole Transcriptome Atlas (DSP-WTA) was used to analyse the transcriptome across three spatial compartments of melanoma in patients treated with ICIs [49]. The developed model identified compartment-specific gene signatures that more accurately predicted treatment response than previously published bulk RNA-seq signatures. The best performance was achieved by an eight-gene S100B signature, suggesting that relevant predictive information may also be encoded in tumour cell-intrinsic gene expression [49]. This conclusion is further supported by single-cell and spatial multiomics studies demonstrating that melanoma cells with a mesenchymal-like phenotype are enriched in patients resistant to ICIs, with their transcriptional programme regulated by TCF4 [50]. These findings indicate that spatial transcriptomic analysis can capture not only features of the immune microenvironment but also intrinsic resistance programmes of tumour cells.

The most advanced approaches to studying the TME are referred to as spatial multiomics. These integrate single-cell data, such as Cellular indexing of transcriptomes and epitopes by sequencing (CITE-seq), with high-dimensional tissue imaging platforms, for example, PhenoCycler (CODEX), enabling simultaneous assessment of cellular phenotype, transcriptomic profiles, and spatial organisation within the tumour [48].

Studies using these methods indicate that response to ICI depends not only on the presence of immune infiltration but also on the organisation of lymphoid aggregates and their influence on T-cell functional states. Quek et al., using single-cell spatial multiomics, showed that resistant tumours were characterised by an “immune-striving” TME, with peritumoural lymphoid aggregates and limited T-cell infiltration into the tumour core [48]. In contrast, favourable response to ICIs was associated with lymphoid aggregates enriched in B-cell signatures, consistent with earlier observations highlighting the role of B-cells and tertiary lymphoid structures in the melanoma TME [14,48]. However, the findings of Quek et al. should be interpreted with caution, as the study included only five patients treated with anti-PD-1/anti-CTLA-4 therapy. These results suggest a potential role of the organisation of lymphoid aggregates, but they do not yet support treating the B-cell signature as a validated predictive biomarker [48].

Despite the small sample size, these observations are consistent with the findings of Cabrita et al. (2020), who used a combination of spatial and transcriptomic approaches to evaluate the role of B-cells and TLS in metastatic melanoma [13]. They demonstrated that the presence of B-cells organised into TLS was associated with improved clinical outcomes, with the longest survival observed in tumours where TLS coexisted with tumour-associated CD8^+^ T-cells. A TLS gene signature predicted outcomes following anti-CTLA-4 and anti-PD-1 therapy in independent cohorts. Tumours enriched in B-cells contained T-cells with a naïve/memory phenotype expressing *TCF7* and *IL7R*, whereas tumours lacking TLS were dominated by a more dysfunctional T-cell phenotype [13].

Consistently, other studies have shown that the presence of TCF7^+^CD8^+^ T-cells is associated with favourable treatment outcomes, whereas higher proportions of PD-1^+^CD38^hi CD8^+^ T-cells correlate with resistance to anti-PD-1 therapy [51]. Taken together with the findings of Quek et al., these results indicate that the relevance of lymphoid aggregates in melanoma depends not only on their presence but also on their organisation, cellular composition, and impact on T-cell functional states [13,48].

### 3.3. Predictive Models Based on H&E Images Beyond Direct Assessment of TIL

Deep learning models applied to routine H&E-stained melanoma specimens are of increasing interest. These approaches learn to recognise morphological patterns in histopathological images related to inflammatory infiltration, stromal architecture or tumour–stroma interactions and, on this basis, predict response to treatment with ICIs, without the need for direct, manual assessment of TILs [30,31,32]. This is particularly important in practice, as H&E slides are widely available for routine diagnosis and their use does not require additional immunohistochemical staining or more complex spatial techniques.

Several independent papers have shown that analysis of H&E images using deep learning models can provide clinically relevant predictive information [30,31,32]. Hu et al. (2021) and Johannet et al. (2021) reported promising early results for predicting response to anti-PD-1 treatment from H&E images, with AUCs of approximately 0.78–0.81 [30,32]. In the study by Hu et al., the deep learning model outperformed the authors’ automated H&E-based TIL assessment, which achieved an AUC of 0.58. However, this result should not be interpreted as general evidence of the limited predictive value of TILs, as the TIL score was based on tile-level classification of H&E images as TIL or non-TIL [30]. This may partly explain the discrepancy with the findings of Wong et al., Fortman et al., and Schuiveling et al., in which digital or immunohistochemical assessment of TILs/CD8 demonstrated a more pronounced predictive value [23,25,26]. The study by Hu et al. therefore suggests that deep learning models may capture additional morphological features that are not reflected in simplified TIL-based assessments [30].

These findings were later confirmed on a larger scale by Fa’ak et al. (2025) [31]. In that study, model performance improved when the analysis was restricted to slides with more tumour tissue and when image-based predictions were combined with clinical data, indicating that the value of deep learning models based on H&E images depends on both data quality and clinical context [31]. The authors also found that favourable treatment outcomes were associated with certain histomorphological features, such as epithelioid morphology, a low tumour-to-stroma ratio and higher TIL infiltration, suggesting that H&E-based models may capture biologically relevant features linked to response to ICIs [31].

In conclusion, these studies suggest that H&E image analysis using deep learning may be a practical complement to more complex TME assessment methods, particularly due to its wide availability and lower cost, although these approaches still require further validation and standardisation [30,31,32].

### 3.4. Predictive Features of the Tumour Microenvironment Across Different ICI Regimens

Many studies employing digital pathology and spatial profiling techniques have evaluated predictors of response to ICIs in melanoma primarily in the context of anti-PD-1 monotherapy or combined anti-PD-1/anti-CTLA-4 treatment regimens [13,23,24,25,26,27,31,32,42,43,48,49].

However, predictive factors should be interpreted within the context of a specific immunotherapy regimen, as these treatment strategies differ in efficacy, toxicity profiles, and mechanisms of action. Consequently, a given biomarker may partly reflect differences between treatment modalities rather than the intrinsic sensitivity of the tumour to immunotherapy [52].

The largest body of evidence from digital pathology and spatial profiling studies concerns anti-PD-1 therapy. In this setting, favourable treatment outcomes have been associated with increased CD8+ TIL infiltration, higher quantitative TIL scores, closer spatial proximity between immune and tumour cells, and immune-hot spatial architectures [23,24,25,26,27,34,42,43,49].

Data regarding anti-CTLA-4 monotherapy are more limited. Nevertheless, TLS- and B-cell-related signatures were associated with improved outcomes in cohorts treated with either anti-CTLA-4 or anti-PD-1 therapies [13]. Similarly, Fa’ak et al. (2025) identified largely comparable morphological features associated with treatment response across both anti-CTLA-4 and anti-PD-1 regimens [31]. However, their model predicted response to nivolumab (anti-PD-1) with an AUC of 0.69, whereas lower performance was observed for ipilimumab (anti-CTLA-4; AUC 0.60) and for combined ipilimumab–nivolumab therapy (AUC 0.61). These differences may have been influenced by characteristics of the analysed material, including histological subtype and biopsy site [31]. Therefore, further studies are required to determine whether distinct predictive features are associated with different ICI treatment strategies when assessed using digital pathology and spatial profiling approaches.

For combined anti-PD-1/anti-CTLA-4 therapy, potential biomarkers of response included increased TIL infiltration, a low tumour-to-stroma ratio, epithelioid tumour morphology, and the presence of organised lymphoid aggregates characterised by a B-cell signature [14,25,31,48].

Data on predictors of response to anti-LAG-3 therapy remain scarce. At present, clinically approved LAG-3 blockade in melanoma is used predominantly in combination with anti-PD-1 therapy [53]. In the study by Pybus et al. (2026), multiplex immunofluorescence (mIF) identified TIL-associated niches in responders to anti-PD-1 therapy that were simultaneously enriched for CD8, LAG-3, PD-L1, and SOX10, suggesting that the predictive value of LAG-3 may depend on its co-occurrence with cytotoxic lymphocytic infiltration and an immunologically active tumour microenvironment [27]. However, a spatial TME profile specifically associated with response to combined anti-PD-1/anti-LAG-3 therapy has not yet been defined.

## 4. Methodological Limitations and Confounding Factors

As discussed in Section 3, digital pathology and spatial methods can provide promising biomarkers for predicting response to ICIs in melanoma patients. However, the predictive value of a biomarker alone does not determine its clinical utility if the result remains sensitive to pre-analytical and analytical factors. Therefore, it is important to determine the conditions under which the results can be considered reliable and comparable between centres. These limitations apply both to WSI analyses based on H&E or IHC and to more complex spatial techniques, where an additional source of variability is the way tissue compartments such as TLS and regions of interest are defined [14,23,24,25,26,27,28,29,30,31,32].

### 4.1. Scanner Variability

As many of the methods described in Section 3 are based on WSI analysis, the way in which slides are digitised remains an important source of technical variability [23,24,31,32]. AI models may lose performance on external test sets due to domain shift, one important component of which is scanner variability [54]. Different scanners generate images with different colour characteristics due to differences in resolution, optics and acquisition parameters [55]. Therefore, validation should include data that are as technically diverse as possible, and results should be reported separately for different scanner types [54].

However, analysis of studies on the prediction of response to ICIs in melanoma patients indicates limited hardware variation. For example, Fortman et al. used the Aperio AT2 scanner [23]; other papers used the ScanScope AT2 [29,31], and Chatziioannou et al. used the Hamamatsu Nanozoomer scanner [24]. Johannet et al. (2021), using the Aperio AT2 or Leica SCN400 scanners, obtained very similar performance in predicting response to ICIs treatment therapy with scanners [32]. However, the lack of systematic robustness tests for scanner variability in most papers suggests that the effectiveness of the models may not be preserved in laboratories with other equipment. Consequently, predictive models may partly reflect technical image features rather than solely tumour biology. A possible solution is physical colour calibration, as proposed by Ji et al., which unifies the appearance of WSI across scanners and improves the generalisability of AI models [55].

### 4.2. Stain Variation in Slides

Similar to hardware variation, differences in slide staining can also affect the stability of H&E-based models. Stain variation includes differences in staining intensity, contrast and colour tones, which may alter the features used by the algorithm. This issue is illustrated by the results of Lin et al., who showed in a study of early non-small-cell lung cancer (NSCLC) that a deep learning model trained on one batch of slides lost its generalisation ability when tested on another batch derived from the same tissue block but stained at a different time [56].

Several studies have attempted to address this issue in the context of predicting response to ICI in melanoma. Hu et al. used the Macenko normalisation method, and Johannet et al. demonstrated the superiority of the Reinhard method over the Vahadane approach and over no normalisation [30,32]. However, Lin et al., using NSCLC samples as an example, showed that conventional stain normalisation and cycle-consistent generative adversarial network (CycleGAN) methods did not clearly improve generalisation between batches, indicating that the problem remains unresolved [56]. This issue is particularly relevant in multicentre studies, where it may limit the comparability of results despite identical model architecture and consistent clinical response criteria, and should also be considered in studies on the prediction of response to ICIs in melanoma patients.

### 4.3. Region of Interest Selection

The selection of the ROI is a key factor influencing the results of pathomorphological studies. Different approaches to defining ROIs may lead to different conclusions even for material from a single patient, as shown in a study assessing human epidermal growth factor receptor 2 (HER2) expression in breast cancer [57]. A similar issue may arise in studies predicting response to ICIs in melanoma, where the choice of ROI can influence the results obtained.

This issue is particularly relevant in studies on quantitative TIL analysis, in which ROIs are often defined manually by experienced pathomorphologists or dermatopathologists [23,24]. As a result, outcomes may partly depend on how precisely the boundaries of the analysed area are delineated.

Furthermore, studies using DSP differ in both ROI definition and tissue compartment segmentation. Toki et al. described ROIs as manually or molecularly defined regions, analysing three compartments: melanocytic, leukocytic and macrophage-related [28]. In contrast, Martinez-Morilla et al. used ROIs of 650 µm in diameter for each core, which were then subdivided into four molecular compartments: tumour, macrophage, leukocyte and non-immune stem cell [29].

In WSI-based deep learning approaches, images are divided into thousands of tiles or patches. Differences in selection procedures and in the proportion of tumour and stromal tissue included in the analysis may affect results, even when the starting material is identical. Therefore, the method of ROI selection should be clearly reported to allow for the proper control of confounding factors [30,31,32]. An attempt at standardisation is the SpatialCells tool, which automatically divides tissue into macroregions based on distance from the tumour boundary, reducing reliance on subjective visual assessment. However, the reliability of the results still depends on the quality of cell segmentation [58].

### 4.4. Heterogeneity of Treatment and Treatment Sequences

Additional limitations may arise from the heterogeneity of clinical cohorts. An important confounding factor is the combined analysis of patients treated with different ICI regimens, such as anti-PD-1 monotherapy, anti-CTLA-4 monotherapy, or combination immunotherapy, as well as patients treated in different lines of therapy [13,23,24,25,26,27,31,32,36,37,42,43,48,49]. In studies employing digital pathology and machine learning, this approach may increase the cohort size but can simultaneously reduce model interpretability, as the model may partly capture differences related to the type of immunotherapy administered rather than true biological determinants of treatment response.

This issue becomes increasingly relevant with the introduction of new therapeutic strategies, such as combined anti-PD-1/anti-LAG-3 therapy. In one study, compared with anti-PD-1 monotherapy, treatment with anti-PD-1 plus anti-LAG-3 was associated with prolonged PFS in patients with low CD8 expression as well as in those with high intratumoural co-expression of CD8 and LAG-3. Although these findings cannot yet be regarded as predictive biomarkers, they support caution when extrapolating biomarkers identified for anti-PD-1 monotherapy to anti-PD-1/anti-LAG-3 combination therapy [37].

Treatment line and prior exposure to ICIs are similarly important, as the immunological state of the TME may differ substantially between immunotherapy-naïve patients and those who have developed resistance following previous ICI treatment. For example, the RELATIVITY-020 study evaluated the expression of multiple biomarkers in patients with advanced melanoma treated with anti-PD-1 and anti-LAG-3 therapy, taking into account prior immunotherapy exposure and the type of resistance to previous treatment [59]. Baseline expression of LAG-3, PD-L1, and CD8, as well as inflammatory gene signatures, varied according to previous immunotherapy, the most recent treatment received, and the type of resistance to prior immunotherapy. Therefore, predictive biomarkers developed in patients initiating immunotherapy should not be automatically extrapolated to patients with prior failure of ICI therapy, and future studies should evaluate these populations separately [59].

### 4.5. Material Collection Site and Sample Type

The biopsy site and type of specimen (e.g., primary tumour, lymph node or soft tissue metastasis) may also act as confounding factors. The immune microenvironment can vary depending on lesion location, affecting both the morphological and spatial features observed in histopathology as well as the efficacy of ICI therapy [60].

Smithy et al. (2025) noted that heterogeneity in lesion location within their retrospective cohort, which included primary tumours, soft tissue or lymph node metastases and visceral metastases, likely reflected differences in tissue microenvironments not captured in the analysis [14]. Similarly, Fa’ak et al. (2025) showed that the performance of an H&E-based model varied depending on biopsy site and histological tumour type [31]. In contrast, Schuiveling et al. included both metastasis location and specimen type directly in the model input, demonstrating differences in TIL rates across sites, with higher rates in lymph nodes and lung and lower rates in the liver [25]. These findings indicate that both specimen type and lesion location should be taken into account in H&E-based studies as well as in more complex spatial analyses.

### 4.6. Heterogeneous Timing of Tissue Collection Versus Treatment

Studies on ICI therapy indicate that the time between tissue collection and the start of immunotherapy may affect the assessment of TME biomarkers. The tumour microenvironment is dynamic and can be altered by prior treatment, particularly in terms of PD-L1 expression [61,62]. Thus, both the time interval and the therapies administered during this period are relevant.

For example, in the study by Smithy et al. (2025) [14], the median interval was 4 months. During this time, 40% of melanoma patients received treatment other than anti-PD-1, and 40% had already received ICI before tissue collection, which the authors considered a significant limitation [14]. A similar limitation was observed in other studies on the prediction of response to ICI based on archival material and retrospective cohorts, making it difficult to interpret biomarkers unambiguously [28,29,49]. This is because a biomarker measured in an archival sample may reflect a prior state of the TME rather than its configuration at the start of ICI therapy. In some studies, tissue was collected before systemic treatment, which partially mitigated this issue [23]. Taken together, these findings indicate that reliable interpretation of biomarkers based on image analysis and spatial data requires careful control of both technical and clinical factors.

## 5. Complementary Biomarkers of Response to Immune Checkpoint Inhibitors

This review focuses primarily on tissue-based approaches, including digital pathology, spatial profiling, and machine learning. However, these approaches should be considered in relation to other biomarkers being developed for the prediction of response to ICIs, such as TMB, the IFN-γ signature, the T-cell-inflamed gene expression profile (GEP), circulating tumour DNA (ctDNA), and selected peripheral blood biomarkers. Each of these parameters reflects a different aspect of disease biology; therefore, their role in predicting response to ICIs should be considered complementary rather than competitive [63,64].

TMB, defined as the number of mutations per megabase of tumour DNA, has been extensively studied as a potential biomarker of response to ICIs. In melanoma, however, its predictive value remains inconclusive, as it may depend on melanoma subtype, mutational status, and inflammatory features of the tumour microenvironment. Analyses of clinical trials have suggested that TMB may be associated with a better treatment response, but its predictive value increases when combined with an inflammatory signature [63]. Similarly, the highest rates of pathological complete response in the OpACIN-neo study were observed among patients with both high TMB and a high IFN-γ signature [65]. This suggests that TMB alone may be insufficient for reliable patient selection but may be useful as part of a predictive model.

The IFN-γ signature and T-cell-inflamed gene expression profile (GEP) reflect T-cell activation, IFN-γ production, antigen presentation, chemokine expression, and cytotoxic activity within the tumour microenvironment. Several studies have demonstrated their association with response to immunotherapy, including pembrolizumab, nivolumab, and combination treatment regimens. However, the presence of an inflamed tumour microenvironment alone does not guarantee ICI efficacy, as a proportion of patients with high IFN-γ signature expression still exhibit treatment resistance [63]. More recent studies have also shown that a serum IFN-γ-related protein signature may predict pathological response to neoadjuvant ICI therapy with comparable accuracy. These findings suggest that biological signals associated with IFN-γ pathway activation may be detectable not only within tumour tissue but also in peripheral blood [66].

Another marker under investigation is ctDNA, assessed by liquid biopsy [67]. High or persistently detectable ctDNA levels during ICI therapy have been associated with higher tumour burden and poorer treatment response, whereas changes in ctDNA levels may enable monitoring of treatment efficacy and early detection of relapse, which is difficult to assess solely on the basis of archival tissue samples [68]. It has also been demonstrated that a significant increase in ctDNA during the early phase of anti-PD-1 treatment may help identify primary resistance in patients with metastatic melanoma and *BRAF* or *KRAS* mutations [69]. Furthermore, in patients treated with ICIs, pretreatment ctDNA levels were associated with first-line treatment outcomes but not with second-line outcomes, particularly in patients previously treated with BRAF/MEK inhibitors, suggesting that the value of ctDNA as a biomarker may depend on prior molecularly targeted therapy [67]. A meta-analysis involving 1063 patients showed that detectable or high ctDNA levels before and during treatment were significantly associated with poorer OS and PFS. These findings support the prognostic value of ctDNA in this patient group, although they do not yet establish ctDNA as a validated predictive biomarker of ICI-specific response [70].

In addition to ctDNA, other peripheral blood biomarkers with potential relevance for predicting ICI efficacy have also been investigated. Several studies have associated better treatment response with normal lactate dehydrogenase (LDH) levels and lower leukocyte-based inflammatory indices, such as the neutrophil-to-lymphocyte ratio (NLR) [64]. Increased cytotoxic activity of CD8+ and CD4+ lymphocytes has also been observed in patients who derive clinical benefit from ICIs. However, the significance of some immune markers, such as sPD-1, sPD-L1, and selected pro-inflammatory cytokines, remains inconclusive and may depend on the type of therapy used. Despite promising findings, the use of blood-derived biomarkers in clinical practice requires further standardisation of sample collection and processing methods, as well as validation in larger studies [64].

In summary, TMB, the IFN-γ signature, T-cell-inflamed GEP, ctDNA, and peripheral immune biomarkers should not be regarded as alternatives to digital pathology and spatial profiling. Their greatest value may lie in their complementary use: TMB reflects the potential immunogenicity of the tumour, gene expression signatures capture local activation of the anti-tumour immune response, ctDNA provides information on tumour burden and disease dynamics, and blood-derived biomarkers reflect the systemic inflammatory and immune response [63,64,67].

## 6. Future Directions

The interpretation of digital and spatial pathomorphology studies in predicting response to ICIs in melanoma remains limited by numerous confounding factors. Future study designs should take into account variability in ROI selection, heterogeneity of treatment regimens and sequences, site of specimen collection and the time between biopsy and initiation of immunotherapy. In studies based on AI-driven H&E image analysis, it is also important to control for scanner variability and staining differences. Standardisation of these elements and their explicit reporting can significantly improve the comparability of results between centres.

Validation of models in external cohorts remains a key issue. Machine learning models are susceptible to overfitting and covariate shift in data distribution, which may lead to overestimation of performance within the training cohort [71]. Therefore, robust external validation and transparent reporting of results are essential. At the same time, much of the existing work is based on small, single-centre cohorts. Multicentre studies, preferably international and involving diverse populations, are needed, particularly as differences in ICI treatment outcomes between ethnic groups have been observed [72]. As a further step towards clinical implementation, well-designed prospective studies with predefined tissue collection time points and clearly defined lines of ICI therapy are required [73].

Future studies should place greater emphasis on the translational and regulatory requirements of digital pathology. Although models for predicting response to ICIs in melanoma remain largely investigational, several AI-based tools in digital pathology have already received regulatory approval or Conformité Européenne (CE) marking for other diagnostic applications. Accordingly, future predictive models should be evaluated not only in terms of performance but also with regard to external validation, regulatory compliance, and applicability in multicentre settings [74,75].

Standardised spatial profiling of large patient cohorts may be facilitated by the Integrated Immunoprofiling of Large Adaptive Cancer Patient Cohorts (IMMUcan) consortium, which employs IFQuant software (available at: https://github.com/BICC-UNIL-EPFL/IFQuant; accessed on 30 May 2026) for the analysis of multiplex immunofluorescence (mIF) data and machine learning algorithms for cell classification in imaging mass cytometry (IMC) datasets [76]. In addition, AI-based platforms such as Lunit SCOPE IO have been used to assess prognostic factors across various solid tumours treated with ICIs and to predict response to ICI therapy in patients with NSCLC [77,78]. These findings provide a rationale for similar studies focused on melanoma. Other platforms used within digital pathology workflows include PathAI and HALO/Indica Labs, which support quantitative whole-slide image (WSI) analysis, immuno-oncology biomarker assessment, and characterisation of the tumour microenvironment. However, their utility as tools for predicting response to ICIs in melanoma remains to be established through dedicated validation studies [79].

These efforts should be complemented by the continued development of AI-based methods for the analysis of routine H&E-stained histopathological images. Routine H&E staining may contain more biological information than is currently used in clinical practice [80]. These approaches are less costly and more accessible than multiplex technologies such as mIF, making them potentially scalable in routine diagnostics. However, many H&E-based models are trained on data paired with spatial methods that provide biological context, and H&E analysis alone does not currently replace high-plex techniques [80]. Further research using methods such as mIF, DSP or IMC is still needed, as they enable simultaneous measurement of multiple biomarkers and analysis of spatial relationships between tumour and immune cells.

Current evidence suggests that single markers, such as TIL count or selected molecular features, may not be sufficient as stand-alone predictive tools [27]. At the same time, digital, quantitative assessment of TIL shows promising results at relatively low cost, supporting further investigation of its clinical value [23,24,25]. However, studies that simultaneously integrate TIL assessment, WSI data and spatial models of the tumour microenvironment remain limited. Combining these levels—including evaluation of inflammatory infiltrates, global tumour architecture and spatial relationships between tumour cells and immune populations—may better reflect the biological complexity of response to immunotherapy and improve the robustness of predictive models.

## 7. Clinical Translation and a Stepwise Implementation Workflow

Despite promising results, digital pathology and spatial profiling should not currently be regarded as standalone tests for selecting patients with melanoma for ICI therapy. A more realistic approach would be a stepwise implementation model, in which these methods serve as tools supporting clinical decision making. The first step could involve AI-assisted analysis of routine H&E-stained slides and selected IHC stains, as these are already part of everyday diagnostic practice. Such analysis could provide quantitative and interpretable data on TILs, CD8+ cell density, the tumour-to-stroma ratio and the spatial distribution of immune cells [23,24,25,33]. The result should be reported as additional information for the pathologist and oncologist, rather than as an autonomous therapeutic recommendation.

Implementation of such a workflow would, however, require appropriate technical and organisational infrastructure. This includes the purchase of a WSI scanner, image storage infrastructure, integration with laboratory information systems, software validation and staff training. The cost of a WSI scanner may range from tens to hundreds of thousands of US dollars, while the cost of a single scan in a large oncology centre may range from USD 0.55 to USD 19.53, depending mainly on the number of slides scanned and scanner utilisation [81].

**Figure 2:**
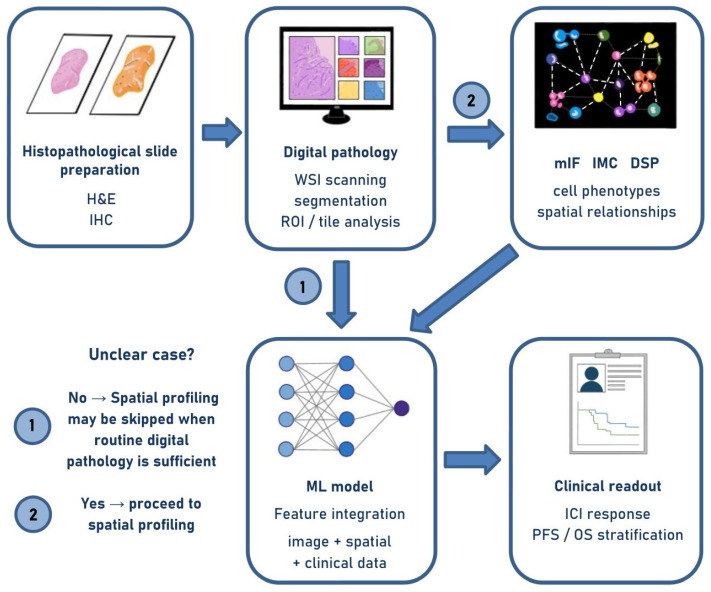
Proposed stepwise workflow for the implementation of digital pathology and spatial profiling in the prediction of response to ICIs in melanoma. Histopathological slides (H&E and IHC) undergo digital pathology analysis, including WSI scanning, tissue segmentation, and ROI/tile-based feature extraction. In cases where routine digital pathology provides sufficient information, image-derived features may be directly integrated into an ML model. In selected or diagnostically ambiguous cases, additional spatial profiling methods (mIF, IMC, and DSP) can be applied to characterise cellular phenotypes and spatial relationships within the tumour microenvironment. Integrated image, spatial, and clinical data are subsequently used for ML-based prediction of treatment response and clinical outcome stratification. Abbreviations: H&E, haematoxylin and eosin; IHC, immunohistochemistry; WSI, whole-slide imaging; ROI, region of interest; mIF, multiplex immunofluorescence; IMC, imaging mass cytometry; DSP, Digital Spatial Profiling; ML, machine learning; ICI, immune checkpoint inhibitor; PFS, progression-free survival; OS, overall survival. All figures were prepared using CollaNote version 4.1.2 and Microsoft Word 2016.

In selected cases, more complex spatial methods, such as mIF, DSP, IMC or spatial transcriptomics, could be applied. Their use would be particularly justified in cases with ambiguous H&E/IHC findings or suspected more complex mechanisms of resistance, as their routine implementation is limited by higher costs and technical complexity [82]. A proposed stepwise workflow for implementing digital pathology and spatial techniques in the prediction of response to ICIs in patients with melanoma is presented in Figure 2.

## 8. Conclusions

Predicting response to immune checkpoint inhibitors in melanoma patients remains a significant clinical challenge. Previous studies indicate that the efficacy of ICIs depends not only on the presence of immune cells within the tumour, but also on their functional state, spatial organisation and relationship with tumour cells and stroma. Therefore, spatial and high-plex techniques, often analysed with artificial intelligence tools and machine learning algorithms, better reflect the actual complexity of the tumour microenvironment than classical single biomarkers.

At the same time, a growing body of data indicates that simpler and more accessible approaches based on quantitative TIL assessment and analysis of routine H&E slides, often supported by AI methods, can show significant predictive value. They have the advantage of using material available in daily diagnostic practice, with lower costs and greater implementation potential than multiplex and spatial profiling methods. The most promising direction therefore seems to be the development of integrated models combining digital analysis of routine histopathology with more advanced spatial characterisation of the TME. However, standardisation of methods, external validation and well-designed prospective studies remain prerequisites for their clinical implementation.

## Data Availability

No new data were created or analyzed in this study. Data sharing is not applicable to this article.

## Abbreviations

The following abbreviations are used in this manuscript:

AUC	Area under the curve
AI	Artificial intelligence
BRAF	B-Raf proto-oncogene, serine/threonine kinase
CAF	Cancer-associated fibroblast
CITE-seq	Cellular indexing of transcriptomes and epitopes by sequencing
CD1a	Cluster of differentiation 1a
CD3	Cluster of differentiation 3
CD4	Cluster of differentiation 4
CD8	Cluster of differentiation 8
CD20	Cluster of differentiation 20
CD68	Cluster of differentiation 68
CD95	Cluster of differentiation 95
CE	Conformité Européenne
CODEX	Co-detection by indexing
cDC1	Conventional type 1 dendritic cells
CTLA-4	Cytotoxic T-lymphocyte-associated protein 4
ctDNA	Circulating tumour DNA
CXCL10	C-X-C motif chemokine ligand 10
CXCL9	C-X-C motif chemokine ligand 9
CXCR3	C-X-C motif chemokine receptor 3
CycleGAN	Cycle-consistent generative adversarial network
DNA	Deoxyribonucleic acid
DSP	Digital spatial profiling
DSP-WTA	Digital spatial profiling whole transcriptome atlas
eTILs	Electronic tumour-infiltrating lymphocytes score
FOXP3	Forkhead box P3
GEP	Gene expression profile
GeoMx	GeoMx Digital Spatial Profiler platform
H&E	Haematoxylin and eosin
CD38^hi	High CD38 expression
HER2	Human epidermal growth factor receptor 2
IMC	Imaging mass cytometry
IMMUcan	Integrated Immunoprofiling of Large Adaptive Cancer Patient Cohorts
ICI	Immune checkpoint inhibitor
IHC	Immunohistochemistry
iNOS	Inducible nitric oxide synthase
IFN-γ	Interferon gamma
IFQuant	Immunofluorescence quantification software
IL-15	Interleukin 15
IL7R	Interleukin 7 receptor
KRAS	Kirsten rat sarcoma viral oncogene homologue
LAG-3	Lymphocyte-activation gene 3
LDH	Lactate dehydrogenase
MHC I	Major histocompatibility complex class I
mregDC	Mature regulatory dendritic cells
mRNA	Messenger ribonucleic acid
MDM	Monocyte-derived macrophage
MEK	Mitogen-activated protein kinase kinase
mIF	Multiplex immunofluorescence
MDSC	Myeloid-derived suppressor cell
NLR	Neutrophil-to-lymphocyte ratio
NSCLC	Non-small-cell lung cancer
OS	Overall survival
PD-1	Programmed cell death protein 1
PD-L1	Programmed death-ligand 1
PFS	Progression-free survival
QIF	Quantitative immunofluorescence
ROI	Region of interest
RNA	Ribonucleic acid
RNA-seq	RNA sequencing
Treg	Regulatory T-cell
S100B	S100 calcium-binding protein B
SOX10	SRY-box transcription factor 10
sPD-1	Soluble programmed cell death protein 1
sPD-L1	Soluble programmed death-ligand 1
TLS	Tertiary lymphoid structures
TCF4	Transcription factor 4
TCF7	Transcription factor 7
TGF-β	Transforming growth factor beta
TIICs	Tumour-infiltrating immune cells
TIL	Tumour-infiltrating lymphocyte
TME	Tumour microenvironment
TMB	Tumour mutational burden
WSI	Whole-slide imaging
